# (D620N) VPS35 causes the impairment of Wnt/β-catenin signaling cascade and mitochondrial dysfunction in a PARK17 knockin mouse model

**DOI:** 10.1038/s41419-020-03228-9

**Published:** 2020-11-30

**Authors:** Ching-Chi Chiu, Yi-Hsin Weng, Ying-Zu Huang, Rou-Shayn Chen, Yu-Chuan Liu, Tu-Hsueh Yeh, Chin-Song Lu, Yan-Wei Lin, Yu-Jie Chen, Chia-Chen Hsu, Chi-Han Chiu, Yu-Ting Wang, Wan-Shia Chen, Shu-Yu Liu, Hung-Li Wang

**Affiliations:** 1 Neuroscience Research Center, Chang Gung Memorial Hospital at Linkou, Taoyuan, Taiwan; 2 Department of Nursing, Chang Gung University of Science and Technology, Taoyuan, Taiwan; 3 Department of Medical Biotechnology and Laboratory Science, Chang Gung University College of Medicine, Taoyuan, Taiwan; 4 Healthy Aging Research Center, Chang Gung University College of Medicine, Taoyuan, Taiwan; 5 Division of Movement Disorders, Department of Neurology, Chang Gung Memorial Hospital at Linkou, Taoyuan, Taiwan; 6 College of Medicine, Chang Gung University, Taoyuan, Taiwan; 7 Institute of Cognitive Neuroscience, National Central University, Taoyuan, Taiwan; 8 Division of Sports Medicine, Landseed International Hospital, Taoyuan, Taiwan; 9 Department of Neurology, Taipei Medical University Hospital, Taipei, Taiwan; 10 Department of Physiology and Pharmacology, Chang Gung University College of Medicine, Taoyuan, Taiwan

**Keywords:** Cell death in the nervous system, Cellular neuroscience

## Abstract

Patients with familial type 17 of Parkinson’s disease (PARK17) manifest autosomal dominant pattern and late-onset parkinsonian syndromes. Heterozygous (D620N) mutation of vacuolar protein sorting 35 (VPS35) is genetic cause of PARK17. We prepared heterozygous VPS35^D620N/+^ knockin mouse, which is an ideal animal model of (D620N) VPS35-induced autosomal dominant PARK17. Late-onset loss of substantia nigra pars compacta (SNpc) dopaminergic (DAergic) neurons and motor deficits of Parkinson’s disease were found in 16-month-old VPS35^D620N/+^ mice. Normal function of VPS35-containing retromer is needed for activity of Wnt/β-catenin cascade, which participates in protection and survival of SNpc DAergic neurons. It was hypothesized that (D620N) VPS35 mutation causes the malfunction of VPS35 and resulting impaired activity of Wnt/β-catenin pathway. Protein levels of Wnt1 and nuclear β-catenin were reduced in SN of 16-month-old VPS35^D620N/+^ knockin mice. Downregulated protein expression of survivin, which is a target gene of nuclear β-catenin, and upregulated protein levels of active caspase-8 and active caspase-9 were observed in SN of VPS35^D620N/+^ mice at age of 16 months. VPS35 is involved in controlling morphology and function of mitochondria. Impaired function of VPS35 caused by (D620N) mutation could lead to abnormal morphology and malfunction of mitochondria. A significant decrease in mitochondrial size and resulting mitochondrial fragmentation was found in tyrosine hydroxylase-positive and neuromelanin-positive SNpc DAergic neurons of 16-month-old VPS35^D620N/+^ mice. Mitochondrial complex I activity or complex IV activity was reduced in SN of 16-month-old VPS35^D620N/+^ mice. Increased level of mitochondrial ROS and oxidative stress were found in SN of 16-month-old VPS35^D620N/+^ mice. Levels of cytosolic cytochrome c and active caspase-3 were increased in SN of VPS35^D620N/+^ mice aged 16 months. Our results suggest that PARK17 mutant (D620N) VPS35 impairs activity of Wnt/β-catenin signaling pathway and causes abnormal morphology and dysfunction of mitochondria, which could lead to neurodegeneration of SNpc DAergic cells.

## Introduction

About 1% of the population older than the age of 60 are affected with Parkinson’s disease (PD), which is the most prevalent neurodegenerative motor disease^1^. PD patients exhibit classical symptoms of motor dysfunction, which include akinesia, bradykinesia, resting tremor, and rigidity. Neuropathological hallmarks underlying PD clinical manifestations are neurodegeneration of substantia nigra pars compacta (SNpc) dopaminergic (DAergic) cells and presence of intracellular Lewy bodies^2^. Most of PD patients (~90%) are sporadic cases, and hereditary genetic mutations cause ~10% of PD cases^1,3–6^. Patients with familial type 17 of Parkinson’s disease (PARK17) manifest autosomal dominant pattern and late-onset parkinsonism syndromes^3,4,6^. In 2011, two research groups^7,8^ used exome sequencing technology to identify mutations of PARK17 in vacuolar protein sorting 35 (VPS35) gene. Heterozygous Asp620Asn (D620N) mutation of VPS35 is a confirmed genetic cause of PARK17 (refs. ^7–10^). PARK17 patients with (D620N) VPS35 mutation displayed cardinal symptoms of PD and exhibited the improvement after L-dopa treatment^7,8^. Interestingly, (D620N) mutation of VPS35 was also observed in sporadic PD patients^11^. Knockin mouse harboring disease-causing gene mutation is an ideal animal model for investigating the pathogenic mechanism of hereditary human disease^12^. Knockin mouse expressing PARK17 (D620N) VPS35 has not been used to study molecular pathogenic mechanisms involved in (D620N) VPS35 mutation-induced loss of SNpc DAergic cells and resulting PD.

VPS35 protein is expressed in various brain areas, such as striatum (ST) and SN^13^. VPS35 is the critical element of retromer multi-subunit complex^14–18^. Retromer is consisted of VPS26–VPS29–VPS35 trimer and sorting nexin (SNX) dimer. Retromer complex controls intracellular trafficking of proteins by associating with endosomes and mediating protein transport from endosomes to trans-Golgi network (TGN) or plasma membrane^14–16,18^. SNX dimer is required for recruiting retromer to endosomes. VPS35–VPS29–VPS26 trimer participates in cargo binding and functions as the cargo recognition complex^14,18^. VPS35-containing retromer complex-mediated protein trafficking regulates activity of several signal transduction pathways, including Wnt signaling cascades^10,17,19^. Wntless promotes Wnt secretion by transporting Wnt proteins from TGN to cell membrane where Wnt ligands are released. VPS35-containing retromer, which mediates the endosome-to-TGN trafficking of Wntless, is required for normal release of Wnt proteins and subsequent activation of Wnt signaling pathways^19–23^. One of Wnt signaling cascades is canonical Wnt/β-catenin pathway^24–26^. Wnt protein plays key roles in development and survival of SNpc DAergic neurons through activating Wnt/β-catenin signaling cascade^27,28^. Wnt receptor Frizzled-1 and β-catenin are found in SNpc DAergic neurons of mouse brain^29^. Wnt1 promotes differentiation of midbrain DAergic neurons and maintains survival of SN DAergic neurons by activating Wnt/β-catenin signaling cascade^30,31^. Wnt1 activation of Wnt/β-catenin cascade prevents 6-hydroxydopamine-induced neurotoxic effect on DAergic neurons^32^. Specific knockout of β-catenin causes the loss of SNpc DAergic neurons, indicating that β-catenin is needed for survival of SNpc DAergic cells^33^. (D620N) VPS35 mutation has been shown to disrupt cargo sorting function of retromer and cause trafficking defects^34^. Therefore, it is very likely that PARK17 mutant (D620N) VPS35 causes a defective secretion of Wnt and an impaired activity of Wnt/β-catenin signaling, leading to cell death of SNpc DAergic neurons and PD^25,35^.

Mitochondria play an essential role in ATP generation, regulation of intracellular calcium level, activation of apoptotic pathway, and oxidative stress response^36,37^. Mitochondrial dysfunction is a major pathogenic mechanism that causes neuronal death of SNpc DAergic cells, and resulting sporadic or familial PD^36–38^. Malfunction of mitochondria is believed to cause an increase in mitochondrial level of ROS and cytochrome c release to cytosol, which leads to induction of mitochondria-mediated apoptotic pathway and loss of SNpc DAergic neurons in PD patients^36,37^. Normal morphology and function of mitochondria is maintained by constant, and dynamic process of fission and fusion^36,37^. Abnormal morphology of mitochondria caused by an impaired process of mitochondrial dynamics results in mitochondrial dysfunction, overproduction of ROS, and subsequent apoptotic death of SNpc DAergic neurons^36,37^. VPS35 deficiency has been shown to cause fragmentation of mitochondria in DAergic neurons by impairing mitochondrial fusion^39^. A previous in vitro study reported that mutant (D620N) VPS35-induced mitochondrial fragmentation and dysfunction by promoting fission process of mitochondria^40^. Further study using (D620N) VPS35 knockin mouse is required to provide in vivo evidence that PARK17 mutant (D620N) VPS35 causes abnormal morphology and malfunction of mitochondria.

Heterozygous (D620N) mutation of VPS35 causes autosomal dominant PARK17 (refs. ^7–10^). In the present study, heterozygous VPS35^D620N/+^ knockin mice were prepared as the animal model of mutant (D620N) VPS35-induced autosomal dominant PARK17. Heterozygous VPS35^D620N/+^ mice displayed late-onset pathological and behavioral phenotypes of PD. Our data suggest that PARK17 mutant (D620N) VPS35 causes cell death of SNpc DAergic neurons via impairing activity of Wnt/β-catenin neuroprotective signaling, and causing abnormal morphology and dysfunction of mitochondria.

## Materials and methods

### Preparation of heterozygous VPS35^D620N/+^ knockin mice

GAT codon for Asp-620 in exon 15 of mouse VPS35 gene was altered to AAT codon of Asn by performing oligonucleotide-directed mutagenesis using PCR amplification. DNA fragment of exon 15 containing (D620N) mutation was ligated into pBluescript vector. LoxP-flanked neomycin selection (Neo) sequence was added into intron 15. Knockin target vector containing (D620N) fragment of exon 15, LoxP-flanked Neo cassette, 5′ and 3′ homologous sequence was transfected into 129/Sv ES cells. PCR assays were conducted to screen neomycin-resistant colonies with correct homologous recombination. ES cells with correct targeting were microinjected into C57BL/6 J blastocysts. Then, chimeric mouse was crossed with wild-type (WT) C57BL/6 J mice. Resulting mutant heterozygous F1 mice were tested for germline transmission by PCR assay. (D620N) VPS35 knockin mutation was confirmed by direct sequencing of exon 15 after PCR amplification of tail DNA. Neo cassette was removed by breeding F1 mice carrying (D620N) VPS35 mutation with EllA-Cre transgenic mice. Heterozygous VPS35^D620N/+^ knockin mice were maintained on C57BL/6 J background. Procedures of animal experiments were approved by Institutional Animal Care and Use Committee (IACUC No. CGU107-166) of Chang Gung University.

### PCR assays of knockin mice expressing mutant (D620N) VPS35

Forward primer (5′-TCTATGTATACCAGGCATTTTCTCTATATG-3′) and reverse primer (5′-AAAGACAACAAGAAGAAATTAGATATCCTG-3′) were used to perform PCR assay using tail DNA of WT or VPS35^D620N/+^ mouse. PCR products for WT allele and VPS35^D620N^ allele were 660 and 710 bp, respectively.

### RT-PCR confirmation of (D620N) VPS35 knockin mutation

Total RNA was purified from SN of WT or VPS35^D620N/+^ mouse by using Trizol Reagent (Invitrogen). The first strand cDNA was synthesized using total RNA extracted from SN of WT or VPS35^D620N/+^ mouse, as described previously^41^. Then, PCR was carried out with cDNA and specific primers of exon 15 of VPS35 gene. PCR product was then gel purified and used for DNA sequencing.

### Behavioral tests

Following behavioral tests were performed to assess motor performance of WT or VPS35^D620N/+^ mice: (1) to measure distance and velocity of locomotion, mice were placed in an open-field behavioral space. Video camera was used to record locomotion of mice for 40 min, which was then quantified using TopScan (Clever Sys.) motion tracking software. (2) Motor performance of WT or VPS35^D620N/+^ mice was determined by conducting pole test. For this test, mice were placed on top of a pole, and the base of pole was put in home cage. Mice descend along the pole and walk to home cage. Following 2 days of training, mice carried out five trials, and the time needed for walking along the pore was recorded.

### Immunohistochemical staining

Following the anesthesia, WT or VPS35^D620N/+^ mice were perfused with 4% paraformaldehyde. Permeabilized coronal sections (20 μm) of frozen brain were interacted with one of following primary antibodies: (1) tyrosine hydroxylase (TH) antibody (Merck Millipore, MAB318); (2) NeuN antiserum (Merck Millipore, MAB377); (3) α-synuclein antibody (Proteintech, 10842-1-AP); (4) phospho-α-synuclein^Ser129^ antiserum (Abcam, ab51253); and (5) phospho-Tau^Ser202/Thr205^ antiserum (Thermo Scientific, MN1020). Following the incubation with biotinylated secondary antibody, brain slices were incubated with avidin-biotin-HRP (Vector Laboratories). As described previously^41^, StereoInvestigator software (MBF Bioscience) was used to quantify the number of TH-positive SN DAergic neurons or NeuN-positive neurons. ImageJ software was utilized to measure optical density of striatal TH staining.

### Immunoblotting study

As described in our previous study^41^, SN tissue was dissected out from coronal brain slices of midbrain under a microscope according to the stereotaxic atlas of mouse brain. CHAPS lysis buffer was used to obtain proteins from SN tissue of WT or VPS35^D620N/+^ mouse. For preparing cytosolic fraction, SN tissues were sonicated in CHAPS buffer containing protease inhibitor cocktail (Sigma). After centrifugation at 12,000 r.p.m., the supernatant, which contains cytosolic fraction, was obtained and used for immunoblotting study. According to manufacturer’s protocol, Mitochondria Isolation Kit (ThermoFisher Scientific) was utilized to isolate mitochondrial proteins from SN tissues of WT or VPS35^D620N/+^ mouse. To collect nuclear fraction, SN tissues were sonicated in lysis buffer (5 mM DTT, 2 mM MgCl_2_, 10 mM Tris, pH = 7.4, and commercial proteinase inhibitor), and homogenates were centrifuged at 2500 r.p.m. Resulting pellet was resuspended in lysis buffer and centrifuged at 2500 r.p.m. Nuclear extract was collected by adding extraction buffer (10% glycerol, 1 mM DTT, 2 mM MgCl_2_, 2 mM EDTA, 0.4 M KCl, 20 mM Tris, pH = 8.0 and commercial proteinase inhibitor) to the pellet. Following centrifugation at 15,000 r.p.m., supernatant was utilized as nuclear extract.

Cytosolic, mitochondrial, or nuclear fractions of SN or protein extracts of SN (30 μg) separated on SDS–PAGE were transferred to nitrocellulose membranes. Then, membranes were hybridized with following primary antibodies: (1) VPS35 antibody (Abcam, ab10099); (2) α-synuclein antiserum (Proteintech, #10842-1-AP); (3) phospho-α-synuclein^Ser129^ antibody (Abcam, ab51253); (4) Tau antiserum (Santa Cruz Biotechnology, sc-32274); (5) phospho-Tau^Ser202,/Thr205^ antiserum (Thermo Scientific, MN1020); (6) Wnt1 antibody (Abcam, ab15251); (7) β-catenin antiserum (Abcam, ab6302); (8) survivin antibody (Abcam, ab76424); (9) cytochrome c antiserum (Abcam, ab133504); (10) cleaved caspase-8 antibody (Cell Signaling Technology, #9496, iREAL Biotechnology, IR99-409); (11) cleaved caspase-9 antiserum (Cell Signaling Technology, #20750); (12) cleaved caspase-3 antibody (Cell Signaling Technology, #9662); (13) mitofusin 2 antiserum (Cell Signaling Technology, #9482); (14) Drp1 antibody (Cell Signaling Technology, #8570); (15) actin antiserum (Abcam, ab8226); (16) histone H3 antibody (Merck, 05-449); and (17) COX IV antiserum (Abcam, ab16056). Immunoreactive proteins on membranes were visualized by interacting with appropriate HRP-conjugated secondary antibody.

### Transmission electron microscopy

The brains of mice were fixed with 4% paraformaldehyde/2.5% glutaraldehyde reagent. SN sections were cut in 1-mm^3^ slices and incubated in 1% osmium tetroxide for 2 h. SN slices were dehydrated with an increasing concentrations of ethanol solutions. Dehydrated SN slices were then embedded in Epon resin (Electron Microscopy Sciences) and sectioned into ultrathin specimens (80 nm). Cellular and mitochondrial structures were visualized using transmission electron microscope (Hitachi HT7800). The mitochondrial size was quantified by using ImageJ software.

### Analysis of mitochondrial complex activity

To measure mitochondrial complex I activity, 30 μg mitochondrial extracts were loaded into 96-well microplate and interacted with anti-complex I monoclonal antibody coated in the microplate. The oxidation of reduced NADH to NAD^+^ caused by mitochondrial complex I leads to an increase in the absorbance at OD 450 nm.

For measuring activity of mitochondrial complex II, mitochondrial fraction (30 μg) was added into microplate wells coated with anti-complex II antibody. Mitochondrial complex II catalyzes ubiquinone to ubiquinol, which couples DCPIP dye (blue color) to DCPIPH2 (colorless). Mitochondrial complex II activity was examined by a decreased OD 600 nm value.

To measure mitochondrial complex III activity, mitochondrial extracts (30 μg) were incubated with complex III activity solution containing oxidized cytochrome c. Mitochondrial complex III converts oxidized cytochrome c to reduced cytochrome c, leading to an increase at OD 550 nm.

For measuring activity of mitochondrial complex IV, 30 μg of mitochondrial fraction was added into microplate coated with anti-complex IV monoclonal antibody. Mitochondrial complex IV activity was evaluated by oxidation of reduced cytochrome c, which resulted in a decrease at OD 550 nm.

### Measurement of intracellular ATP

ATP Colorimetric/Fluorometric Assay Kit (BioVision) was utilized to examine the content of intracellular ATP. Mitochondrial extract (20 μl) was added into 96-well plate and interacted with reaction mix of kit. After incubation for 30 min, intracellular ATP level was colorimetrically determined by measuring OD 570 nm.

### Measurement of mitochondrial respiration

SN tissues of WT or VPS35^D620N/+^ mice were loaded into XFe24 microplate (Agilent). Then, mitochondrial respiration was determined by measuring mitochondrial oxygen consumption rate with aid of Seahorse XFe24 Analyzer (Agilent).

### Analysis of ROS

The level of ROS was evaluated by utilizing OxiSelect In Vitro ROS/RNS assay kit (Cell Biolabs). A total of 50 μl of mitochondria extracts were applied into microplate and interacted with catalyst reagent (50 μl) of kit. DCFH-DiOxyQ, the ROS probe, was added into mixture and oxidized to fluorescent DCF in the presence of ROS. Microplate detector was utilized to determine fluorescent intensity of DCF at 480 nm (excitation)/530 nm (emission).

### Determination of mitochondrial lipid peroxidation

TBARS (thiobarbituric acid reactive substances) assay kit (Cayman Chemicals) was utilized to evaluate mitochondrial lipid peroxidation of SN tissue from WT or VPS35^D620N/+^ mouse. Thiobarbituric acid binds to malondialdehyde, final metabolite of lipid peroxidation, and generates thiobarbituric acid–malondialdehyde adduct. Mitochondrial fractions or malondialdehyde standards were incubated with thiobarbituric acid, and the level of malondialdehyde was then examined by measuring OD 540 nm.

### Colorimetric determination of caspase-9 or caspase-3 activity

Colorimetric caspase-9 or caspase-3 assay kit (Abcam) was used to analyze the activity of caspase-9 or caspase-3 in SN of WT or VPS35^D620N/+^ mice. Active caspase-9 and active caspase-3 recognize the LEHD and DEVD sequence, respectively. Active caspase cleaves chromophore *p*-nitroaniline- or *p*-nitroanilide-labeled substrates, and releases chromophore *p*-nitroaniline or *p*-nitroanilide. The activity of caspase-9 or caspase-3 was evaluated by determining OD 400 nm.

### Statistics

Unpaired Student’s *t* test was utilized to assess statistical significance between two groups. One-way ANOVA with Tukey test was utilized to analyze multiple groups. The *P* value <0.05 was considered as significant.

## Results

### Heterozygous VPS35^D620N/+^ knockin mice exhibit late-onset loss of SNpc DAergic neurons

**Fig. 1:**
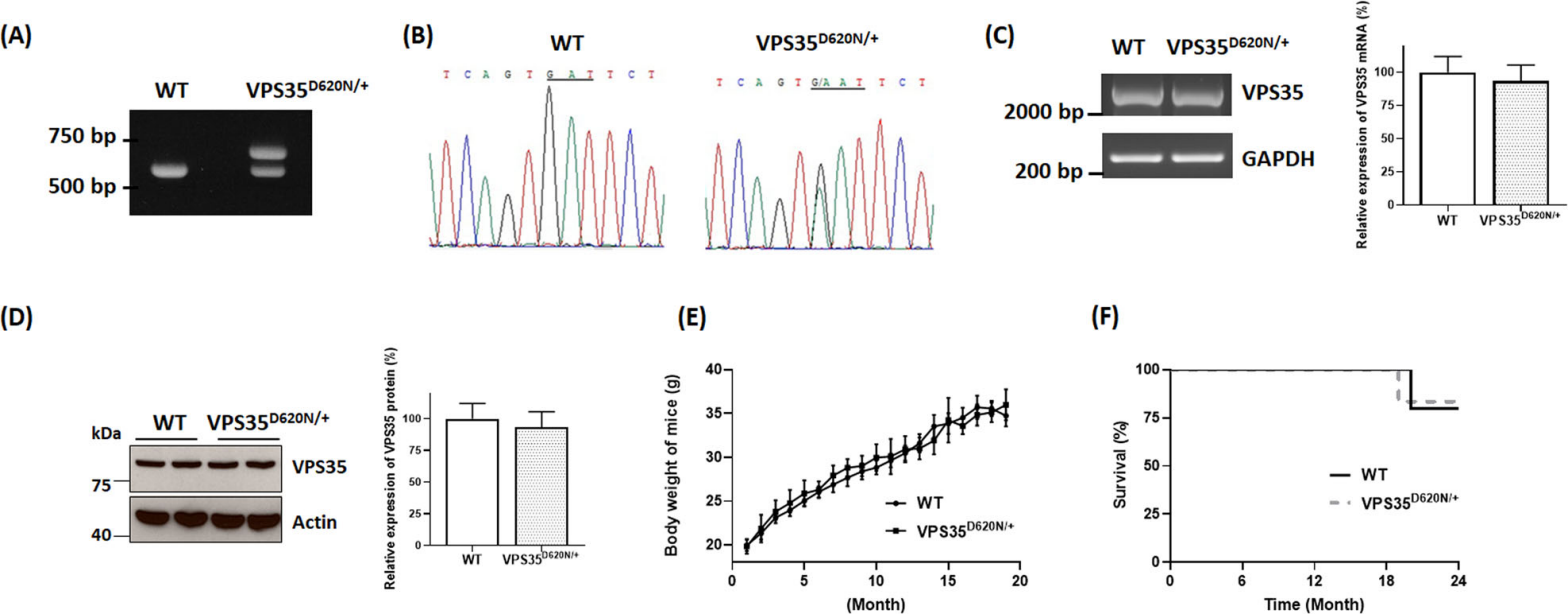
Protein expression of VPS35 is not altered in SN of heterozygous VPS35^D620N/+^ knockin mouse. **A** Heterozygous VPS35^D620N/+^ mouse was identified by performing PCR assays using tail DNA of wild-type (WT) or VPS35^D620N/+^ mouse. Expected sizes of PCR products for WT allele and VPS35^D620N^ allele were 660 and 710 bp, respectively. **B** Following RT-PCR reaction using total RNA extracted from SN of WT or VPS35^D620N/+^ mouse, DNA sequencing showed that in contrast to normal GAT (D620) sequence of VPS35 in WT mouse, both mutated AAT (N620) and wild-type GAT (D620) sequences of VPS35 were found in heterozygous VPS35^D620N/+^ mouse. **C** Following RT-PCR reaction, a similar level of mRNA, which encodes full-length coding region of VPS35 mRNA, was observed in SN of WT or VPS35^D620N/+^ mouse. Real-time quantitative RT-PCR assays showed that VPS35 mRNA level in SN of VPS35^D620N/+^ knockin mice was not significantly different from that of WT mice. Each bar shows mean ± S. E. value of four mice. **D** Protein level of VPS35 in SN of VPS35^D620N/+^ knockin mouse was similar to that of VPS35 in SN of WT mouse. Each bar represents mean ± S. E. value of five mice. **E** WT and VPS35^D620N/+^ mice exhibited similar body weight–age curves. Each point shows mean ± S. E. value of 30–35 mice. **F** WT mice and heterozygous VPS35^D620N/+^ mice displayed similar Kaplan–Meier survival curves (WT mice, *n* = 30; VPS35^D620N/+^ mice, *n* = 30).

Heterozygous (D620N) mutation of VPS35 is a confirmed genetic cause of autosomal dominant PARK17 (refs.^7–10^). Therefore, we generated heterozygous VPS35^D620N/+^ knockin mice (Fig. 1A) as mouse model of mutant (D620N) VPS35-induced autosomal dominant PARK17. VPS35^D620N/+^ knockin mouse was prepared by performing target vector-mediated homologous recombination and introducing (D620N; GAT → AAT) mutation in exon 15 of VPS35 gene. Total RNA was extracted from SN of WT or VPS35^D620N/+^ knockin mice. Subsequent RT-PCR reaction and direct DNA sequencing were performed to verify heterozygous (D620N) mutation of VPS35 mRNA in VPS35^D620N/+^ knockin mouse (Fig. 1B). PCR reactions using genomic DNA of WT or VPS35^D620N/+^ mouse were conducted to obtain DNA fragments of VPS35 exons and their adjacent introns. Subsequent DNA sequencing indicated that except (D620N; GAT → AAT) mutation in exon 15, sequences of exons and adjacent introns of VPS35 gene in VPS35^D620N/+^ knockin mouse were not altered (Supplementary Table 1 and Supplementary Fig. 1). RT-PCR analysis showed that a similar level of mRNA, which encodes full-length coding region of VPS35 mRNA, was found in SN of WT or VPS35^D620N/+^ mice (Fig. 1C; *n* = 4 mice). Real-time quantitative RT-PCR assays also demonstrated that VPS35 mRNA level in SN of VPS35^D620N/+^ knockin mice was similar to that of WT mice (Fig. 1C). Immunoblotting assays indicated that a similar expression of VPS35 protein was found in SN of WT or VPS35^D620N/+^ mice (Fig. 1D). Breeding with VPS35^D620N/+^ mice produced WT mice, heterozygous knockin mice and homozygous knockin mice with an expected Mendelian ratio. Similar to WT mice, heterozygous VPS35^D620N/+^ mice exhibited normal body weight and survival rate (Fig. 1E, F).

**Fig. 2:**
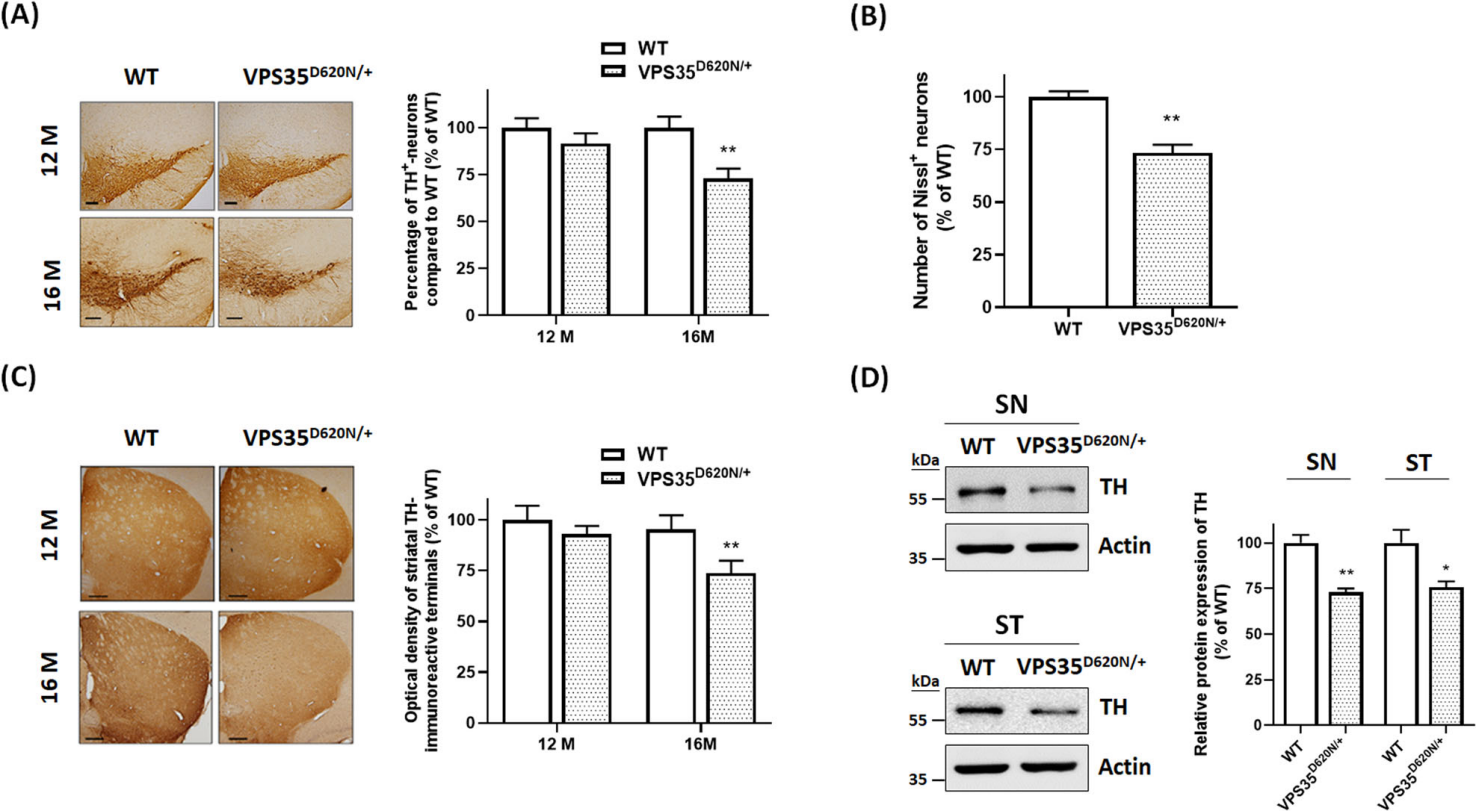
Sixteen-month-old heterozygous VPS35^D620N/+^ knockin mice exhibit late-onset cell death of SNpc DAergic neurons and degeneration of DAergic nigrostriatal terminals. **A** TH staining showed that 12-month-old VPS35^D620N/+^ knockin mice did not display a decrease in number of SNpc DAergic neurons. Compared to WT mice at age of 16 months, age-matched heterozygous VPS35^D620N/+^ mice exhibited the reduction in number of SNpc DAergic neurons. Each bar represents mean ± S. E. value of 11 mice. **P < 0.01 compared with WT mice. **B** Sixteen-month-old VPS35^D620N/+^ mice exhibited a reduction in number of Nissl^+^ cells in SNpc. Each bar shows mean ± S.E. value of eight mice. **C** Compared to WT mice aged 16 months, age-matched VPS35^D620N/+^ knockin mice exhibited the reduction in density of striatal TH immunoreactivity. Each bar represents mean ± S.E. value of 11 mice. **D** Protein level of TH was significantly decreased in SN and striatum (ST) of VPS35^D620N/+^ knockin mice at age of 16 months. Each bar shows mean ± S.E. value of four mice. *P < 0.05 compared with WT mice.

We performed immunohistochemical staining of TH-positive SNpc DAergic neurons using WT or VPS35^D620N/+^mice aged 12 or 16 months. Number of TH-positive SNpc DAergic neurons of 12-month-old VPS35^D620N/+^ knockin mice was similar to that of age-matched WT mice (Fig. 2A). Consistent with previous studies demonstrating that heterozygous (D620N) VPS35 mutation causes late-onset PD^7,8^, heterozygous VPS35^D620N/+^ mice at age of 16 months display a decrease in number of TH-positive SNpc DAergic neurons (Fig. 2A). Number of Nissl^+^ cells was significantly decreased in SNpc of VPS35^D620N/+^ knock mice aged 16 months (Fig. 2B). Loss of SNpc DAergic neurons observed in VPS35^D620N/+^ mice aged 16 months should result in reduction of DAergic nigrostriatal terminals. Compared to WT mice at age of 16 months, level of striatal TH immunoreactivity was decreased in age-matched VPS35^D620N/+^ knockin mice (Fig. 2C). In accordance with results of TH immunohistochemical staining, protein expression of TH was downregulated in SN and ST of 16-month-old VPS35^D620N/+^ mice (Fig. 2D). Immunocytochemical staining of neuronal marker NeuN indicated that reduction of neuronal number was not observed in the ST or cerebral cortex of 16-month-old heterozygous VPS35^D620N/+^ mice (data not shown; *n* = 5 mice).

### Heterozygous VPS35^D620N/+^ knockin mice exhibit late-onset motor impairments of PD

**Fig. 3:**
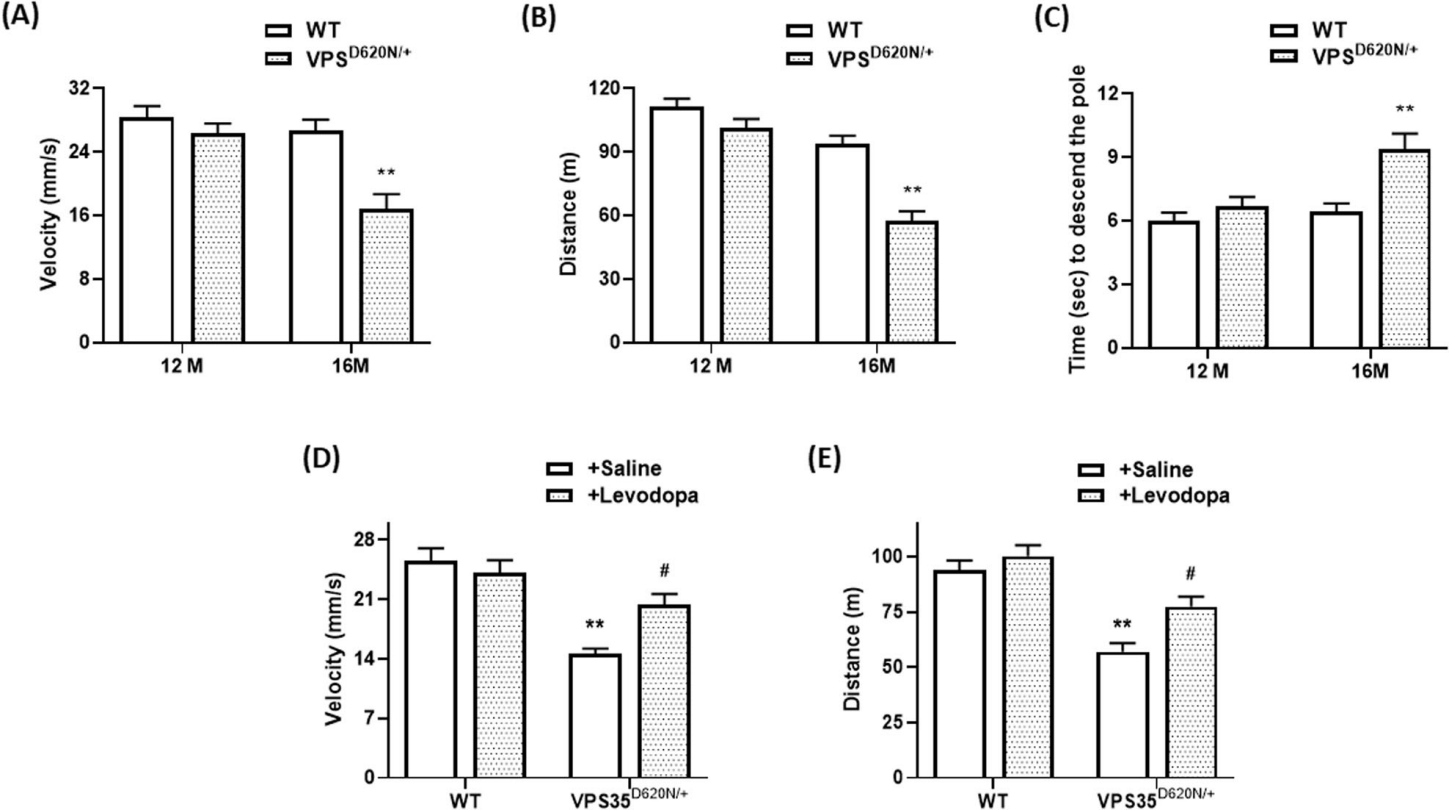
Sixteen-month-old VPS35^D620N/+^ knockin mice exhibit late-onset motor dysfunction of PD. **A**, **B** Motor activity of heterozygous VPS35^D620N/+^ mice aged 12 months was similar to that of WT mice. Velocity (**A**) and distance (**B**) of locomotor activity were significantly reduced in 16-month-old VPS35^D620N/+^ knockin mice. Each bar represents mean ± S.E. value of 11 mice. **P < 0.01 compared with WT mice. **C** Twelve-month-old WT or VPS35^D620N/+^mice performed pole test with a similar time required to descend the pole. VPS35^D620N/+^ knockin mice aged 16 months displayed bradykinesia symptom by requiring more time to perform pole test. Each bar shows mean ± S.E. value of 11 mice. **D**, **E** Forty minutes after intraperitoneal application of saline or methyl L-DOPA (1.5 mg/kg/body weight), locomotor activity was measured. Treatment of methyl L-DOPA significantly improved reduced distance and velocity of locomotion exhibited by VPS35^D620N/+^ knockin mice at age of 16 months. Each bar represents mean ± S.E. value of eight animals. ^#^*P* < 0.05 compared to VPS35^D620N/+^ mice injected with saline.

Mutant (D620N) VPS35-induced loss of SNpc DAergic neurons and impaired DAergic neurotransmission in the ST is expected to cause motor dysfunction, and resulting parkinsonism behavioral phenotypes, including hypokinesia and bradykinesia, of heterozygous VPS35^D620N/+^ mice. The locomotor activity of 12-month-old VPS35^D620N/+^ knockin mice was similar to that of WT mice (Fig. 3A, B). Sixteen-month-old VPS35^D620N/+^ knockin mice displayed late-onset hypokinesia phenotype, and reduced velocity and distance of locomotor activity (Fig. 3A, B). Motor function of WT or VPS35^D620N/+^ mouse was also examined by conducting the pole test. VPS35^D620N/+^ or WT mice aged 12 months exhibited a similar time required to carry out pole test (Fig. 3C). Sixteen-month-old heterozygous VPS35^D620N/+^ mice displayed bradykinesia phenotype and required more time to finish pole test (Fig. 3C). L-DOPA treatment ameliorates PD symptoms exhibited by PARK17 patients with heterozygous (D620N) VPS35 mutation^7,8^. Therefore, L-DOPA is expected to exert a beneficial effect on hypokinesia phenotype displayed by VPS35^D620N/+^ knockin mice. Intraperitoneal application of methyl L-DOPA significantly reversed reduced distance and velocity of locomotion displayed by heterozygous VPS35^D620N/+^ mice aged 16 months (Fig. 3D, E).

### Protein level of α-synuclein or phospho-tau^Ser202/Thr205^ is increased in SN of heterozygous VPS35^D620N/+^ knockin mice aged 16 months

**Fig. 4:**
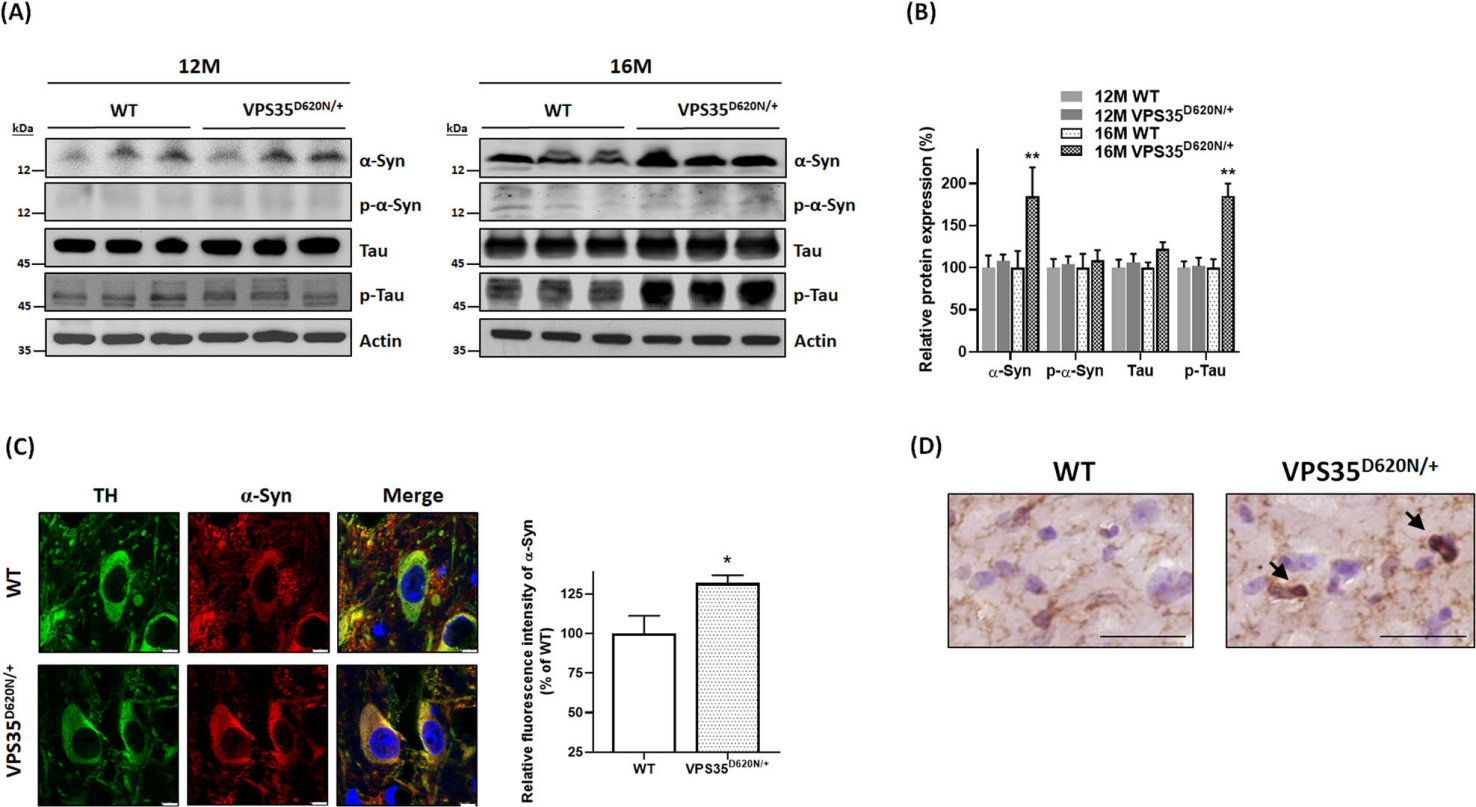
Upregulated levels of α-synuclein and phospho-tau^Ser202/Thr205^ were observed in SN of VPS35^D620N/+^ knockin mice at age of 16 months. **A**, **B** Protein expression of cytosolic α-synuclein (α-Syn) or phospho-tau^Ser202/Thr205^ (p-Tau) was upregulated in SN of 16-month-old heterozygous VPS35^D620N/+^ mice. Upregulated expression of α-synuclein or phospho-tau^Ser202/Thr205^ was not found in SN of VPS35^D620N/+^ mice at age of 12 months. A similar protein level of cytosolic phospho-α-synuclein^Ser129^ (p-α-Syn) or tau was found in SN of 12- or 16-month-old WT and VPS35^D620N/+^ mice. **P < 0.01 compared with WT mice. Each bar represents mean ± S.E. value of seven mice. **C** Confocal double immunofluorescence staining showed that cytosolic expression of α-synuclein was significantly upregulated in TH-positive SNpc DAergic neurons of VPS35^D620N/+^ knockin mice at age of 16 months. *P < 0.05 compared with WT mice. Each bar represents mean ± S.E. value of 80 neurons from four mice. **D** Immunocytochemical staining using anti-phospho-tau^Ser202/Thr205^ antiserum showed that tau pathology-like protein aggregates of phospho-tau (arrow) were found in SN of VPS35^D620N/+^ mice aged 16 months. Scale bar is 30 μm.

Except cell death of SNpc DAergic neurons, another neuropathological finding of PD is the presence of intracellular Lewy bodies^1^, which contain α-synuclein and phosphorylated α-synuclein. Protein level of cytosolic α-synuclein was not significantly altered in SN of 12-month-old VPS35^D620N/+^ knockin mice (Fig. 4A, B). Protein expression of cytosolic α-synuclein was upregulated in SN of VPS35^D620N/+^ knockin mice at age of 16 months (Fig. 4A, B). Confocal immunofluorescence imaging study demonstrated that compared to WT mice, cytosolic level of α-synuclein was significantly increased in TH-positive SNpc DAergic neurons of VPS35^D620N/+^ mice aged 16 months (Fig. 4C). Level of cytosolic phospho-α-synuclein^Ser129^ was not altered in 12- or 16-month-old heterozygous VPS35^D620N/+^ mice (Fig. 4A, B). Immunocytochemical analysis using α-synuclein antiserum or phospho-α-synuclein^Ser129^ antibody indicated that Lewy body was absent in brain areas, including SN, hippocampus, and cerebral cortex, of 16-month-old VPS35^D620N/+^ knockin mice (data not shown; *n* = 5 mice).

Tau pathology, which is believed to be caused by hyperphosphorylation of microtubule-associated protein tau, is observed in patients affected with sporadic or familial PD^42,43^. A similar protein expression of cytosolic tau was observed in SN of WT and VPS35^D620N/+^ mice aged 12 or 16 months (Fig. 4A, B). Level of cytosolic phospho-tau^Ser202/Thr205^ was upregulated in SN of 16-month-old heterozygous VPS35^D620N/+^ mice (Fig. 4A, B). Immunohistochemical staining using anti-phospho-tau^Ser202/Thr205^ antibody demonstrated that tau pathology-like protein aggregates of phospho-tau were found in SN of VPS35^D620N/+^ knockin mice at age of 16 months (*n* = 3 mice; Fig. 4D).

### Activity of Wnt/β-catenin signaling pathway is impaired in SN of VPS35^D620N/+^ knockin mice aged 16 months

**Fig. 5:**
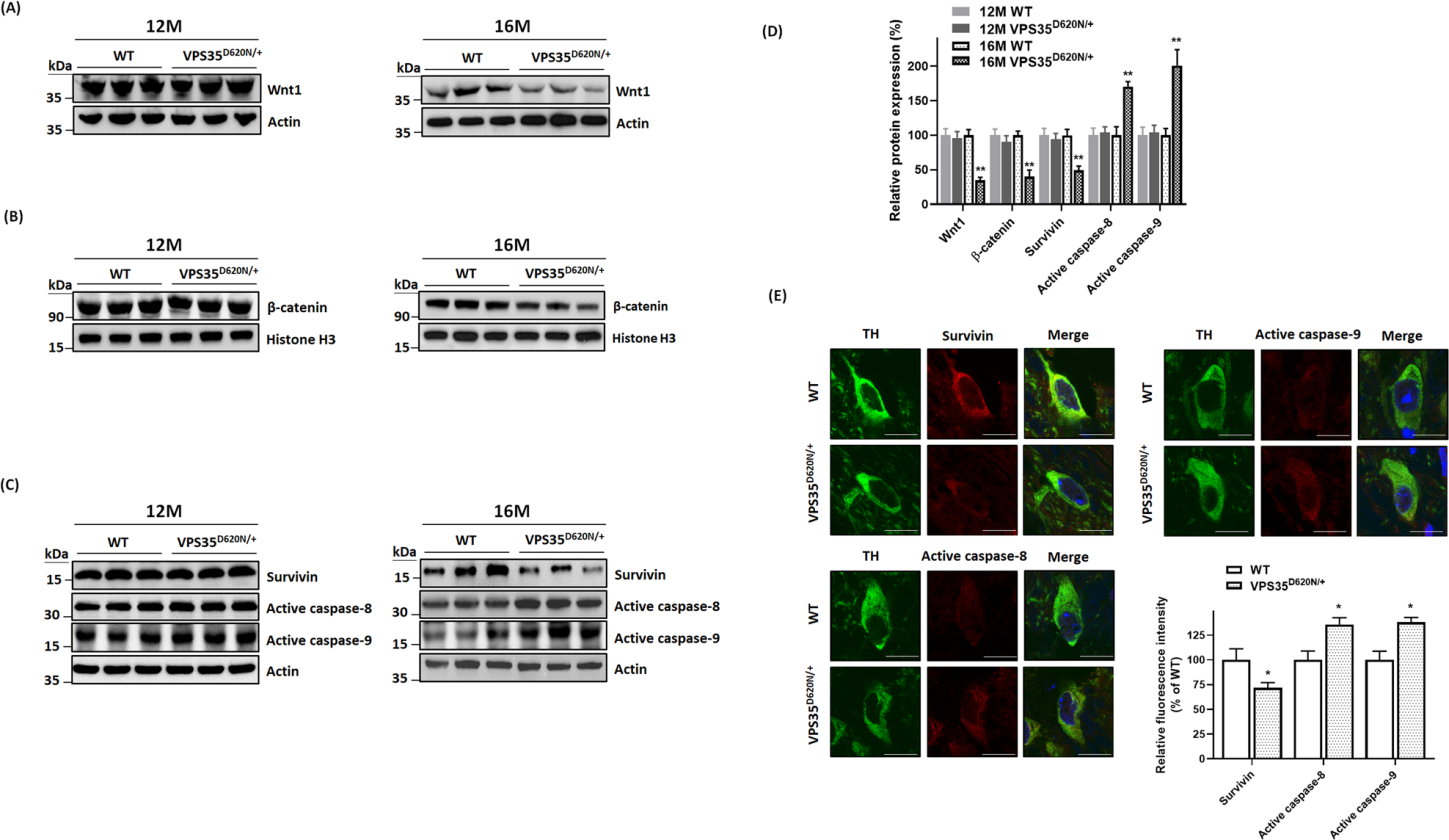
Impaired activity of Wnt/β-catenin signaling cascade and activation of caspase-9 or caspase-8 are found in SN of heterozygous VPS35^D620N/+^ mice aged 16 months. **A**, **D** Protein level of Wnt1 was decreased in SN of VPS35^D620N/+^ knockin mice at age of 16 months. **B**, **D** Protein expression of nuclear β-catenin was downregulated in SN of 16-month-old heterozygous VPS35^D620N/+^ mice. Histone H3 is a protein marker of nuclear fraction. **C**, **D** Western blot study indicated that level of cytosolic survivin was decreased in SN of 16-month-old VPS35^D620N/+^ knockin mice. Cytosolic protein expression of active caspase-9 or active caspase-8 was upregulated in SN of heterozygous VPS35^D620N/+^ mice aged 16 months. Altered expression of Wnt1, nuclear β-catenin, survivin, active caspase-9, or active caspase-8 was not observed in SN of 12-month-old VPS35^D620N/+^ mice. **P < 0.01 compared with WT mice. Each bar represents mean ± S.E. value of seven mice. **E** Confocal immunofluorescence staining showed that downregulated expression of cytosolic survivin and upregulated expression of cytosolic active caspase-8 or caspase-9 were observed in TH-positive SNpc DAergic neurons of VPS35^D620N/+^ knockin mice at age of 16 months. *P < 0.05 compared with WT mice. Each bar represents mean ± S.E. value of 80 neurons from four mice.

In this study, it was hypothesized that mutant (D620N) VPS35 causes the dysfunction of VPS35-containing retromer complex, which results in an impaired secretion of Wnt ligand and subsequent degradation of Wnt protein. Consistent with our hypothesis, protein level of Wnt1, which activates canonical Wnt/β-catenin pathway^24^, was decreased in SN of 16-month-old heterozygous VPS35^D620N/+^ mice (Fig. 5A, D).

Wnt1 activates the neuroprotective signaling cascade and promotes survival of SNpc DAergic neurons by increasing nuclear level of β-catenin^24,30,31^. Nuclear β-catenin is the most important downstream effector protein of Wnt/β-catenin signaling cascade, and level of nuclear β-catenin reflects activity of this pathway^24,26^. Downregulated protein expression of Wnt1 is expected to impair activity of Wnt/β-catenin signaling cascade by decreasing protein level of nuclear β-catenin. Protein level of nuclear β-catenin was reduced in SN of 16-month-old VPS35^D620N/+^ knockin mice (Fig. 5B, D).

Nuclear β-catenin promotes the survival of neurons by upregulating expression of pro-survival and antiapoptotic protein survivin^44,45^, which belongs to inhibitor-of-apoptosis (IAP) family of proteins^46^. Mutant (D620N) VPS35-induced decrease in protein level of nuclear β-catenin is expected to downregulate protein expression of survivin. Immunoblotting assays demonstrated that level of cytosolic survivin was decreased in SN of heterozygous VPS35^D620N/+^ mice aged 16 months (Fig. 5C, D). Confocal double immunofluorescence staining showed that compared to WT mice, downregulated expression of cytosolic survivin was found in TH-positive SNpc DAergic neurons of 16-month-old VPS35^D620N/+^ knockin mice (Fig. 5E). Protein level of Wnt1, nuclear β-catenin, or survivin was not significantly altered in SN of VPS35^D620N/+^ mice at age of 12 months (Fig. 5A–D).

Survivin exerts neuroprotective and antiapoptotic effect by interacting with XIAP, and inhibiting activation of caspase-9 and caspase-8 (ref. ^46^). Downregulated expression of antiapoptotic survivin caused by mutant (D620N) VPS35 could upregulate production of active caspase-8 and active caspase-9. Western blot study indicated that cytosolic protein level of active caspase-9 and active caspase-8 was upregulated in SN of VPS35^D620N/+^ knockin mice aged 16 months (Fig. 5C, D). Protein expression of active caspase-8 or active caspase-9 was not altered in SN of 12-month-old VPS35^D620N/+^ mice (Fig. 5 C, D). Confocal immunofluorescence staining demonstrated that protein expression of active caspase-8 or active caspase-9 was upregulated in TH-positive SNpc DAergic neurons of VPS35^D620N/+^ mice aged 16 months (Fig. 5E).

### Abnormal morphology of mitochondria and mitochondrial dysfunction are found in SN of 16-month-old VPS35^D620N/+^ knockin mice

**Fig. 6:**
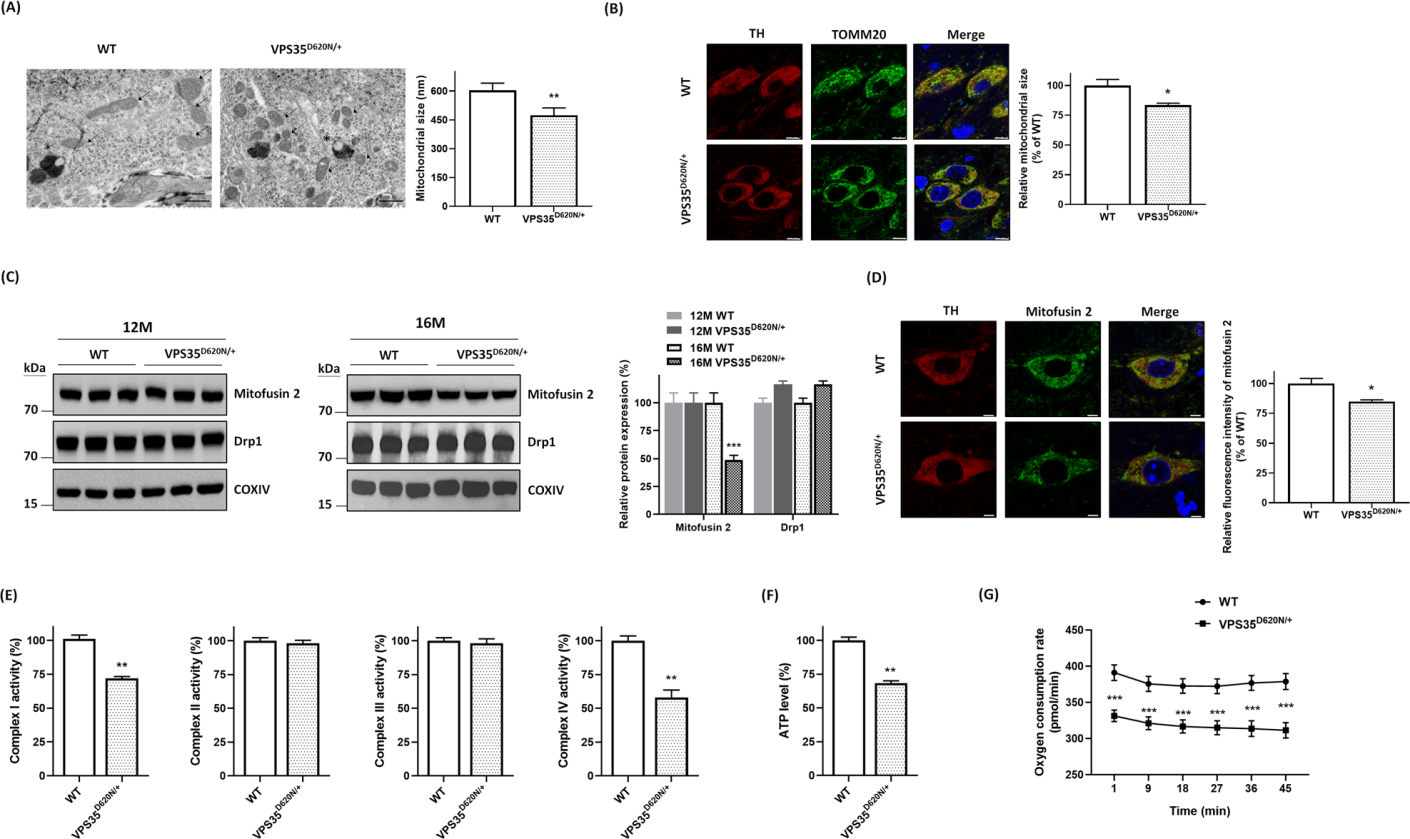
Heterozygous (D620N) mutation of VPS35 causes abnormal morphology and dysfunction of mitochondria. **A** In contrast to intact morphology of mitochondria observed in neuromelanin-positive SNpc DAergic cells of age-matched WT mice, fragmented mitochondria with a reduced size were found in neuromelanin organelle-containing SNpc DAergic neurons of heterozygous VPS35^D620N/+^ mice aged 16 months. Arrow and star indicate mitochondria and neuromelanin organelle, respectively. Each bar represents mean ± S.E. value of 200 mitochondria. **P < 0.01 compared with WT mice. **B** Confocal immunofluorescence staining of Tom20 showed that mitochondria in TH-positive SNpc DAergic neurons of WT mice exhibited a long thread-like structure. In contrast, truncated and shortened mitochondria were found in TH-positive SNpc DAergic neurons of VPS35^D620N/+^ mice at age of 16 months. Each bar shows mean ± S.E. value of 80 neurons from four mice. *P < 0.05 compared with WT mice. **C** Immunoblotting assays demonstrated that protein expression of mitochondrial mitofusin 2 was downregulated in SN of VPS35^D620N/+^ knockin mice aged 16 months. Downregulated expression of mitofusin 2 was not found in SN of 12-month-old VPS35^D620N/+^ mice. Level of mitochondrial Drp1 was not altered in SN of VPS35^D620N/+^ mice at age of 12 or 16 months. COX IV is a protein marker of mitochondrial fraction. Each bar shows mean ± S.E. value of six mice. ***P < 0.001 compared with WT mice. **D** Confocal double immunofluorescence staining demonstrated that protein expression of mitofusin 2 was downregulated in TH-positive SNpc DAergic neurons of VPS35^D620N/+^ knockin mice aged 16 months. Each bar represents mean ± S.E. value of 80 neurons from four mice. **E** Mitochondrial complex I or complex IV activity was reduced in SN of 16-month-old heterozygous VPS35^D620N/+^ mice. Each bar represents mean ± S.E. value of six mice. **F** Intracellular level of ATP was decreased in SN of VPS35^D620N/+^ knockin mice at age of 16 months. Each bar shows mean ± S.E. value of six mice. **G** Compared to WT mice, reduced basal oxygen consumption rate was observed in SN of 16-month-old VPS35^D620N/+^ mice. Each bar represents mean ± S.E. value of three mice.

VPS35 participates in controlling morphology and function of mitochondria by regulating fission or fusion process of mitochondria^10,15,39,40^. Impaired function of VPS35 caused by (D620N) mutation could lead to abnormal morphology of mitochondria in SNpc DAergic neurons. Transmission electron microscopy study demonstrated that compared to normal morphology of mitochondria found in neuromelanin organelle-containing putative SNpc DAergic cells of age-matched WT mice, a significant reduction in the size of mitochondria and resulting mitochondrial fragmentation was observed in neuromelanin-positive SNpc DAergic neurons of heterozygous VPS35^D620N/+^ mice aged 16 months (Fig. 6A). Confocal immunofluorescence staining of Tom20, a mitochondrial marker, was conducted to visualize mitochondrial morphology of TH-positive SNpc DAergic neurons. Mitochondria displayed a normal long thread-like structure in TH-positive SNpc DAergic neurons of WT mice (Fig. 6B). Consistent with results of transmission electron microscopy study, fragmented mitochondria, which are truncated and shortened, were observed in TH-positive SNpc DAergic neurons of 16-month-old VPS35^D620N/+^ mice (Fig. 6B).

VPS35 deficiency has been shown to downregulate protein level of mitofusin 2 (ref. ^39^), which is required for mitochondrial fusion. An in vitro study reported that mutant (D620N) VPS35 caused dysregulated expression of dynamin-related protein 1 (Drp1)^40^, which is needed for mitochondrial fission. Protein expression of mitochondrial mitofusin 2 was not altered in SN of VPS35^D620N/+^ knockin mice at age of 12 months (Fig. 6C). Protein level of mitochondrial mitofusin 2 was decreased in SN of 16-month-old VPS35^D620N/+^ knockin mice (Fig. 6C). A similar expression of mitochondrial Drp1 was found in SN of WT or VPS35^D620N/+^ mice aged 12 or 16 months (Fig. 6C). Confocal immunofluorescence staining showed that compared to WT mice, protein expression of mitofusin 2 was significantly downregulated in TH-positive SNpc DAergic neurons of 16-month-old VPS35^D620N/+^ knockin mice (Fig. 6D).

Mutant (D620N) VPS35-induced abnormal morphology of mitochondria could cause the malfunction of mitochondria and impair activity of mitochondrial complex I–IV. Mitochondrial complex I and IV activities were reduced in SN of 16-month-old heterozygous VPS35^D620N/+^ mice (Fig. 6E). A similar activity of mitochondrial complex II or complex III was observed in SN of WT or VPS35^D620N/+^ mice aged 16 months (Fig. 6E). As a result of mitochondrial dysfunction, a significant reduction of intracellular ATP level was observed in SN of 16-month-old VPS35^D620N/+^ knockin mice (Fig. 6F). Mutant (D620N) VPS35-induced mitochondrial dysfunction also led to the reduced basal oxygen consumption rate and impaired mitochondrial respiration in SN of VPS35^D620N/+^ mice aged 16 months (Fig. 6G).

### Overgeneration of mitochondrial ROS, oxidative stress, and activation of mitochondrial apoptotic cascade are observed in SN of heterozygous VPS35^D620N/+^ mice aged 16 months

**Fig. 7:**
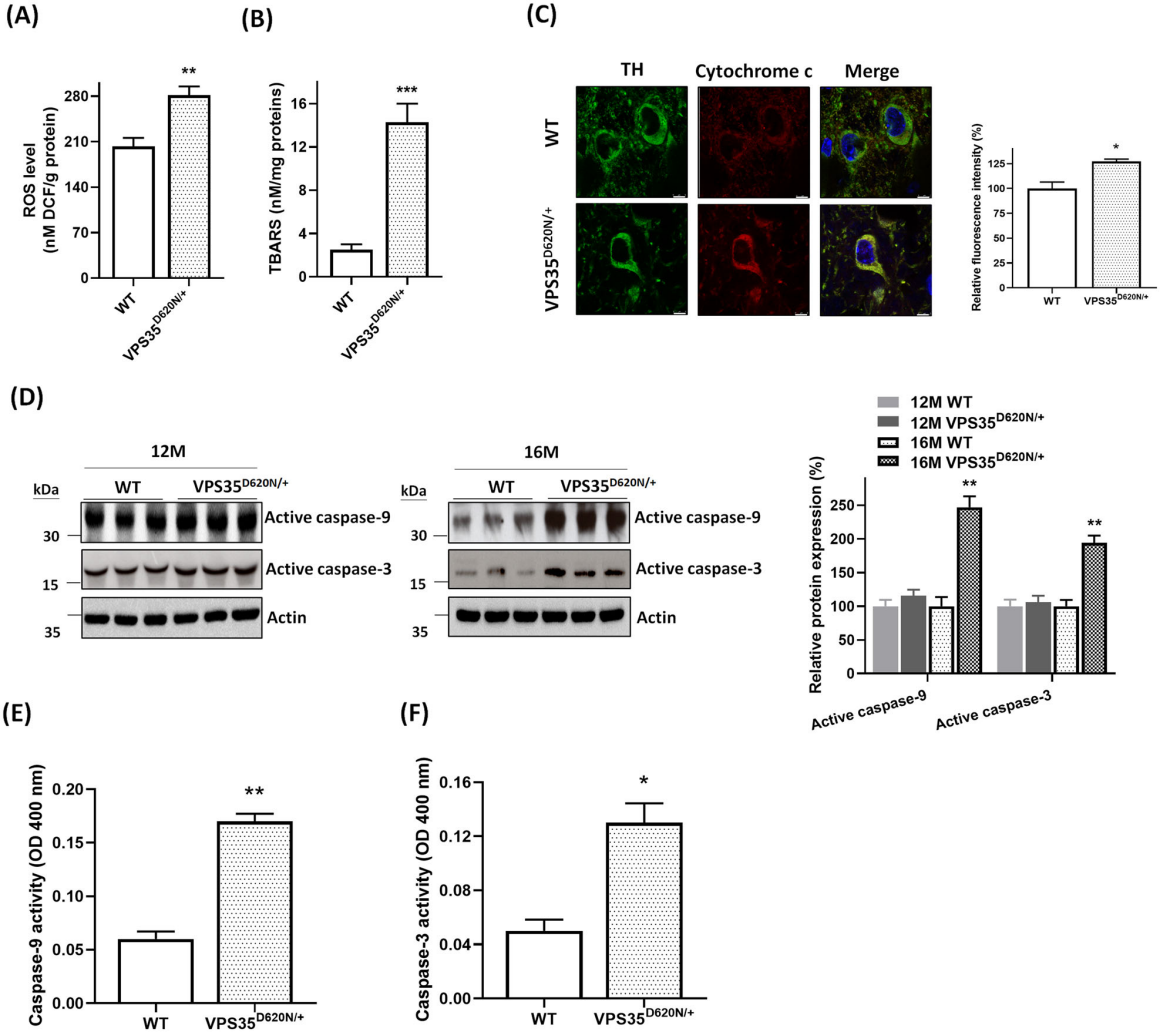
Heterozygous (D620N) mutation of VPS35 causes overgeneration of mitochondrial ROS, oxidative stress, and activation of mitochondria-mediated apoptotic pathway. **A** Level of mitochondrial ROS was upregulated in SN of heterozygous VPS35^D620N/+^ mice aged 16 months. Each bar represents mean ± S.E. value of six mice. **P < 0.01 compared with WT mice. **B** Compared to WT mice, lipid peroxidation of mitochondria was markedly enhanced in SN of 16-month-old VPS35^D620N/+^ knockin mice. Each bar shows mean ± S.E. value of six mice. ***P < 0.001 compared to WT mice. **C** Confocal double immunofluorescence staining demonstrated that protein expression of cytosolic cytochrome c was significantly upregulated in SNpc DAergic neurons of VPS35^D620N/+^ mice aged 16 months. Each bar represents mean ± S.E. value of 80 neurons from four mice. *P < 0.05 compared with WT mice. **D** Cytosolic protein levels of active caspase-9 and active caspase-3 were increased in SN of heterozygous VPS35^D620N/+^ mice aged 16 months. Each bar represents mean ± S.E. value of seven mice. **E** and **F** The activity of caspase-9 or caspase-3 was significantly increased in SN of VPS35^D620N/+^ knockin mice at age of 16 months. Each bar shows mean ± S.E. value of four mice.

Mutant (D620N) VPS35-induced impairment of mitochondrial function is expected to enhance generation of mitochondrial ROS and lipid peroxidation of mitochondria, which results in causing oxidative stress. An upregulated formation of mitochondrial ROS was found in SN of VPS35^D620N/+^ knockin mice at age of 16 months (Fig. 7A). TBARS assay was performed to evaluate lipid peroxidation of mitochondria. A pronounced increase in mitochondrial lipid peroxidation was observed in SN of 16-month-old heterozygous VPS35^D620N/+^ mice (Fig. 7B).

Overgeneration of mitochondrial ROS-induced oxidative stress is believed to cause cytochrome c release from mitochondria to cytosol and activation of mitochondria-mediated apoptotic pathway^36,37^. Confocal immunofluorescence staining showed that protein level of cytosolic cytochrome c was significantly increased in TH-positive SNpc DAergic neurons of heterozygous VPS35^D620N/+^ mice aged 16 months (Fig. 7C). Protein levels of cytosolic active caspase-9 and active caspase-3 were increased in SN of 16-month-old VPS35^D620N/+^ knockin mice (Fig. 7D). Protein expression of active caspase-9 or active caspase-3 was not significantly altered in SN of VPS35^D620N/+^ mice aged 12 months (Fig. 7D). Consistent with results of western blot analysis, activity of caspase-9 (Fig. 7E) or caspase-3 (Fig. 7F) was significantly upregulated in SN of VPS35^D620N/+^ knockin mice at age of 16 months.

## Discussion

Patients with PARK17 display autosomal dominant inheritance^3–8^. Heterozygous (D620N) mutation of VPS35 has been confirmed as the genetic cause of PARK17 (refs. ^7,8^). To better understand molecular pathogenic pathways by which mutant (D620N) VPS35 causes neurodegeneration of SNpc DAergic cells and resulting PD symptoms, we generated heterozygous VPS35^D620N/+^ knockin mouse, which is an ideal animal model of (D620N) VPS35-induced autosomal dominant PARK17. In accordance with previous studies indicating that heterozygous (D620N) mutation of VPS35 causes late-onset PARK17 (refs. ^3,5,7,8^), heterozygous VPS35^D620N/+^ knockin mice aged 16 months exhibited late-onset loss of SNpc DAergic neurons and decrease in density of TH-positive nigrostriatal DAergic terminals. As a result, 16-month-old VPS35^D620N/+^ knockin mice displayed late-onset hypokinesia, which was indicated by a decrease in locomotor velocity or locomotive distance, and bradykinesia, which was demonstrated by a longer time needed to carry out pole test. Consistent with clinical studies demonstrating that L-DOPA treatment exerts a beneficial effect on PARK17 patients carrying (D620N) VPS35 mutation^7,8^, intraperitoneal injection of methyl L-DOPA significantly ameliorated hypokinesia phenotype displayed by heterozygous VPS35^D620N/+^ mice at age of 16 months.

In accordance with our finding, late-onset neurodegeneration of SNpc DAergic cells was also observed in (D620N) VPS35 knockin mice prepared by another group^47^. The loss of DAergic axon terminals in the ST of VPS35^D620N/+^ knockin mice, which is indicated by a reduction in the density of striatal TH staining, is expected to result in an impairment of evoked dopamine release from nigrostriatal DAergic terminals. Consistent with this hypothesis, previous in vivo microdialysis study reported that evoked dopamine release was significantly impaired in the caudate putamen of 5–7-month-old (D620N) VPS35 knockin mice^48^. On the other hand, an augmented dopamine release from striatal slices was observed in 3-month-old (D620N) VPS35 knockin mice^49^.

Up to now, postmortem neuropathological examination has not been conducted to visualize the formation of Lewy bodies, which contain α-synuclein and phosphorylated α-synuclein, in the brain of PARK17 patients with (D620N) VPS35 mutation. Our immunocytochemical staining study indicated that Lewy bodies were not found in brain regions of VPS35^D620N/+^ knockin mice aged 16 months. Interestingly, upregulated level of cytosolic α-synuclein was observed in SN of VPS35^D620N/+^ mice aged 16 months. One of important pathways that participate in the degradation of α-synuclein is chaperone-mediated autophagy^50–52^. Lamp2a is the lysosomal membrane protein for CMA and required for CMA-mediated degradation of α-synuclein. VPS35, a critical component of retromer multi-subunit complex^14–17^, is believed to mediate endosome-to-TGN retrieval of Lamp2a and maintain normal Lamp2a level^52^. (D620N) VPS35 mutation impairs cargo sorting function of retromer and causes trafficking defects^34^. It is very likely that defective trafficking and reduced protein level of Lamp2a caused by mutant (D620N) VPS35 results in impairment of CMA-mediated degradation of α-synuclein, and increased level of α-synuclein in SN of 16-month-old heterozygous VPS35^D620N/+^ mice.

Accumulation of hyperphosphorylated microtubule-associated protein tau could cause tau pathology, which is observed in patients affected with sporadic or hereditary PD^42,43^. Interestingly, increased protein level of phospho-tau^Ser202/Thr205^ was found in SN of VPS35^D620N/+^ mice aged 16 months. Tau pathology-like protein aggregates of phospho-tau^Ser202/Thr205^ were observed in SN of 16-month-old VPS35^D620N/+^ knockin mice. Future study is needed to clarify molecular mechanism underlying mutant (D620N) VPS35-induced phosphorylation of tau. Consistent with our result, tau pathology was also found in the brain of (D620N) VPS35 knockin mice prepared by others^47^. Hyperphosphorylated tau is believed to depolymerize microtubules and cause neuronal dysfunction^42^. The possible involvement of upregulated phospho-tau^Ser202/Thr205^ level in mutant (D620N) VPS35-induced degeneration of SNpc DAergic neurons remains to be studied.

Several lines of evidence indicate that Wnt protein is involved in promoting development and survival of SNpc DAergic neurons through activating Wnt/β-catenin pathway^27–33^. Wnt1 activates canonical Wnt/β-catenin signaling pathway, and facilitates differentiation and survival of SN DAergic neurons^30,31^. Normal expression of β-catenin is required for survival and maintenance of SNpc DAergic neurons^33^. Autocrine or paracrine secretion of Wnt ligands is needed for normal activity of Wnt-mediated pathways. VPS35-containing retromer complex plays a vital role in normal Wnt secretion by recycling Wntless, which mediates release of Wnt, from endosomes to TGN^19–23^. Therefore, normal function of VPS35 is required for Wnt secretion and Wnt/β-catenin signaling activity. In this study, we hypothesized that (D620N) VPS35 mutation causes malfunction of VPS35-containing retromer, which results in a defective secretion of Wnt ligand and subsequent degradation of Wnt protein. In accordance with this hypothesis, level of Wnt1 protein was downregulated in SN of heterozygous VPS35^D620N/+^ mice aged 16 months. Wnt1 promotes survival of SNpc DAergic neurons by increasing protein level of nuclear β-catenin^24,30,31^, which indicates activity of Wnt/β-catenin cascade^24,26^. Downregulated level of Wnt1 protein led to the impairment of Wnt/β-catenin pathway by significantly decreasing nuclear β-catenin in SN of VPS35^D620N/+^ knockin mice at age of 16 months. Nuclear β-catenin exerts a neuroprotective effect by increasing expression of pro-survival and antiapoptotic protein survivin^44,45^. Downregulated expression of nuclear β-catenin caused a significant decrease of cytosolic survivin in SN of 16-month-old VPS35^D620N/+^ mice. Survivin belongs to IAP family of proteins and induces an antiapoptotic and neuroprotective effect by inhibiting activation of caspase-9 and caspase-8^46^. Decreased protein level of cytosolic survivin resulted in upregulated levels of active caspase-9 and active caspase-8 in SN of heterozygous VPS35^D620N/+^ mice at age of 16 months. Our results suggest that mutant (D620N) VPS35 causes degeneration of SNpc DAergic neurons and resulting PARK17 by impairing activity of Wnt/β-catenin neuroprotective signaling cascade. Consistent with our finding, dysregulated Wnt/β-catenin signaling has also been implicated in pathogenesis of sporadic PD^53^.

Dynamic process of mitochondrial fusion and fission regulates normal morphology and function of mitochondria^36,37,54^. VPS35 is involved in controlling mitochondrial dynamics and morphology by regulating process of mitochondrial fission or fusion^10,15,39,40,55^. PARK17 (D620N) mutation-induced dysfunction of VPS35 could result in abnormal morphology of mitochondria in SNpc DAergic neurons. Consistent with this hypothesis, a significant decrease in mitochondrial size and resulting mitochondrial fragmentation was found in SNpc DAergic neurons of 16-month-old VPS35^D620N/+^ knockin mice. A previous study reported that VPS35 deficiency could cause mitochondrial fragmentation in DAergic neurons by decreasing protein level of mitofusin 2 and impairing mitochondrial fusion^39^. In vitro studies suggested that mutant (D620N) VPS35 interacts with Drp1 and causes dysregulated expression of Drp1, which leads to augmented fission process and mitochondrial fragmentation^40,55^. Therefore, mitochondrial fragmentation observed in SNpc DAergic neurons of VPS35^D620N/+^ mice could result from downregulated expression of mitofusin 2 or upregulated expression of Drp1. Immunoblotting assay showed that protein level of mitochondrial mitofusin 2 was decreased in SN of heterozygous VPS35^D620N/+^ mice at age of 16 months and that Drp1level was not altered in SN of VPS35^D620N/+^ mice. Interestingly, downregulated protein expression of mitofusin 2 was also observed in frontal cortex and hippocampus of Alzheimer’s disease patients^56^.

Mutant (D620N) VPS35-induced mitochondrial fragmentation is expected to cause dysfunction of mitochondria. As a result, impaired mitochondrial complex I or complex IV activity, reduced intracellular ATP level and impaired mitochondrial respiration were observed in SN of heterozygous VPS35^D620N/+^ mice aged 16 months. Consistent with our finding, mitochondrial complex I activity was reduced in fibroblasts obtained from a PARK17 patient with (D620N) VPS35 mutation^57^. Impairment of mitochondrial function and complex I activity led to the mitochondrial ROS overgeneration and oxidative stress, which was indicated by increased mitochondrial ROS level and lipid peroxidation in SN of 16-month-old VPS35^D620N/+^ knockin mice. Upregulated generation of mitochondrial ROS and oxidative stress caused activation of mitochondria-mediated apoptotic cascade by increasing cytosolic level of cytochrome c, active caspase-9 or active caspase-3 in SN of 16-month-old heterozygous VPS35^D620N/+^ mice. Our findings suggest that PARK17 mutant (D620N) VPS35 causes neurodegeneration of SNpc DAergic cells by causing mitochondrial fragmentation and dysfunction of mitochondria, which leads to overproduction of mitochondrial ROS and activation of mitochondrial apoptotic pathway.

In summary, we prepared animal model of autosomal dominant and late-onset PARK17 by generating heterozygous VPS35^D620N/+^ knockin mice, which exhibit late-onset neurodegeneration of SNpc DAergic cells and motor dysfunction phenotypes of parkinsonism. The results of our study suggest that PARK17 mutant (D620N) VPS35 induces degeneration of SNpc DAergic neurons by impairing the activity of Wnt/β-catenin signaling cascade, and causing abnormal morphology and malfunction of mitochondria.

## Supplementary information

Supplementary Table 1

Supplementary Figure 1
