# Supplementary material for: (D620N) VPS35 causes the impairment of Wnt/β-catenin signaling cascade and mitochondrial dysfunction in a PARK17 knockin mouse model
Source: Cell Death Dis. 2020 Nov 30;11(11):1018. doi: 10.1038/s41419-020-03228-9 (PMC7705022; doi:10.1038/s41419-020-03228-9)
Supplement: Supplementary file 1 — Supplementary Table 1 [file 41419_2020_3228_MOESM1_ESM.docx]

**Supplementary Table 1. Primers for mouse VPS35 gene**

| Primers | Product size | Primer sequence (5’→3’) |
| --- | --- | --- |
| VPS35 Exon 1 | 189 bp | F: TAAGTGGGCCAATCGAAAAG |
|  |  | R: GAAAACGTGGACTGCGACTC |
| VPS35 Exon 2 | 541 bp | F: ACAGGTTTCTTTGATGCCAGA |
|  |  | R: GTGACACGACTGACGGAGAG |
| VPS35 Eonx 3 | 564 bp | F: GCATGCCTACTTTCTTGACTGC |
|  |  | R: ATCTACCTGCGTTCCAGGACA |
| VPS35 Exon 4 | 436 bp | F: **GTTTTGCCAATGGTGGAAAT** |
|  |  | R: **GGCAAGAAATGCAAGTGTGA** |
| VPS35 Exon 5 | 504 bp | F: **GAGGGGACTGACCACTGAAA** |
|  |  | R: **ACACAACTTACTGACACCCATCA** |
| VPS35 Exon 6 | **465** bp | F: **GAAGCATGAGTGGTTGTGGA** |
|  |  | R: **TGAATCTCTAATCCGTAAAGATGG** |
| VPS35 Exon 7-8 | **977** bp | F: **CATTGAAGTATGGATGAAAGCAA** |
|  |  | R: **TCACGCTTAAGTCAGGCAATC** |
| VPS35 Exon 9-10 | **763** bp | F: **TGCCAATGGAATAAGGCATC** |
|  |  | R: **TCTAACTCCCTCCCCAACAA** |
| VPS35 Exon 11 | **590** bp | F: **ACATGGCCAGTTCTCTGATTCT** |
|  |  | R: **TGAAAAGAGGTATGCCAAATGTT** |
| VPS35 Exon 12 | **400** bp | F: **TTGTACCAATGGGCGAATTT** |
|  |  | R: **TCATTAAGCGTGAGGAAAGGTT** |
| VPS35 Exon 13 | **554** bp | F: **ACCCGTGTAAGCTTCCAATTT** |
|  |  | R: **TGATTCAGACCCTAAGGAAACG** |
| VPS35 Exon 14 | 678 bp | F: TGCGTTCTATAAATGAGGGAAA |
|  |  | R: TTCGCAGCTACCCACTAGTAAAT |
| VPS35 Exon 15 | 580 bp | F: TGAAAGGTACACCGTGCACTA |
|  |  | R: CAACAGTTGCTGTAAACATAGCC |
| VPS35 Exon 16 | 606 bp | F: ACACTCGGCACCAGTTCTGT |
|  |  | R: ATGGACAATGCCCTTAGGAA |
| VPS35 Exon 17 | 531 bp | F: TGAAAGTAATGCAAGTTCCTTAAAA |
|  |  | R: GCCAGAATAAGTGGGTTTTGC |
