# Supplementary figures and images for: (D620N) VPS35 causes the impairment of Wnt/β-catenin signaling cascade and mitochondrial dysfunction in a PARK17 knockin mouse model
Source: Cell Death Dis. 2020 Nov 30;11(11):1018. doi: 10.1038/s41419-020-03228-9 (PMC7705022; doi:10.1038/s41419-020-03228-9)

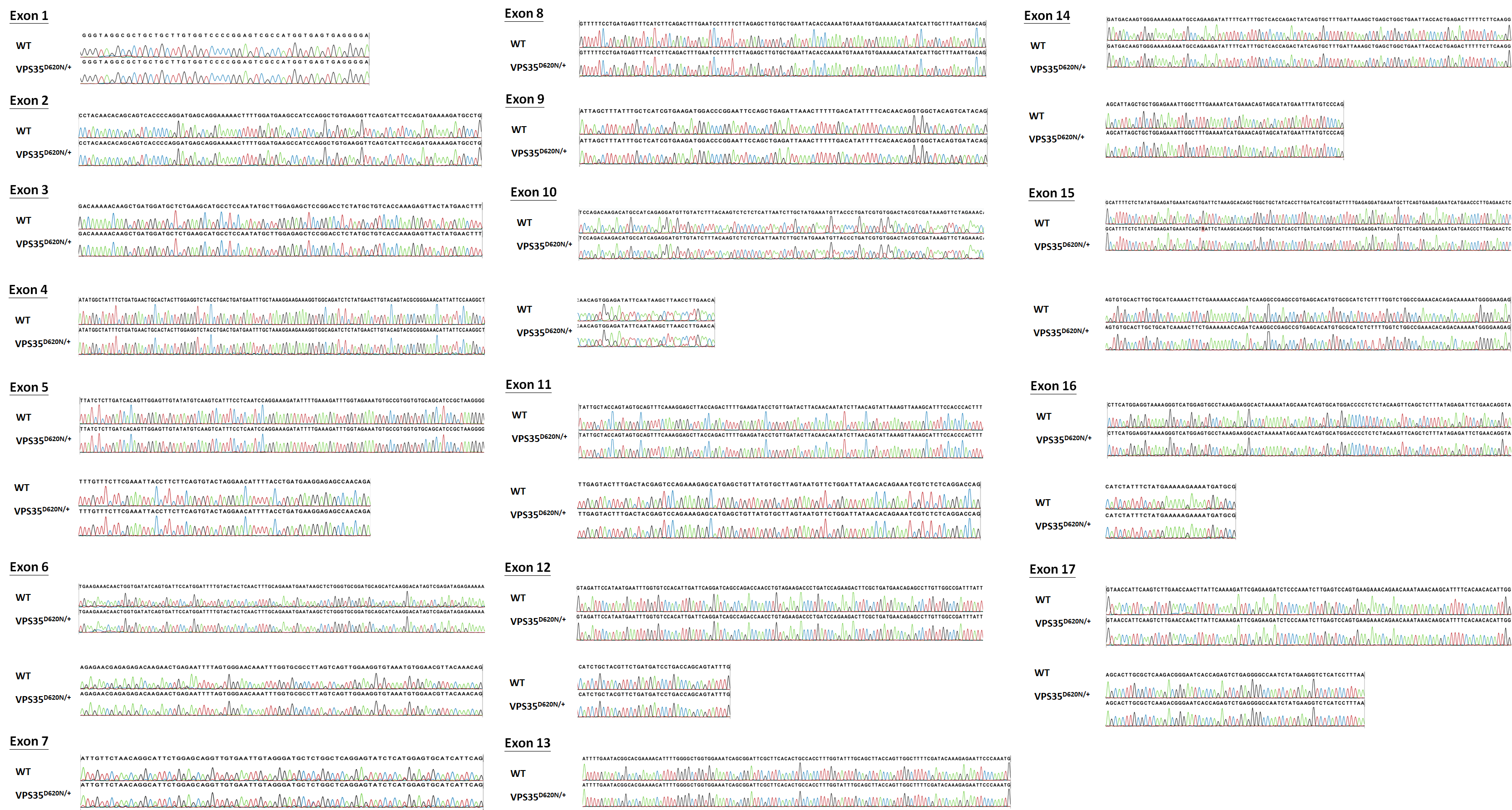

Supplement: Supplementary file 2 — Supplementary Figure 1 [file 41419_2020_3228_MOESM2_ESM.tif]
